# NIR-II fluorescence imaging using indocyanine green nanoparticles

**DOI:** 10.1038/s41598-018-32754-y

**Published:** 2018-09-27

**Authors:** Rohan Bhavane, Zbigniew Starosolski, Igor Stupin, Ketan B. Ghaghada, Ananth Annapragada

**Affiliations:** Department of Pediatric Radiology, Texas Children’s Hospital, Houston, TX 77030 USA

## Abstract

Fluorescence imaging in the second near-infrared window (NIR-II) holds promise for real-time deep tissue imaging. In this work, we investigated the NIR-II fluorescence properties of a liposomal formulation of indocyanine green (ICG), a FDA-approved dye that was recently shown to exhibit NIR-II fluorescence. Fluorescence spectra of liposomal-ICG were collected in phosphate-buffered saline (PBS) and plasma. Imaging studies in an Intralipid^®^ phantom were performed to determine penetration depth. *In vivo* imaging studies were performed to test real-time visualization of vascular structures in the hind limb and intracranial regions. Free ICG, NIR-I imaging, and cross-sectional imaging modalities (MRI and CT) were used as comparators. Fluorescence spectra demonstrated the strong NIR-II fluorescence of liposomal-ICG, similar to free ICG in plasma. *In vitro* studies demonstrated superior performance of liposomal-ICG over free ICG for NIR-II imaging of deep (≥4 mm) vascular mimicking structures. *In vivo*, NIR-II fluorescence imaging using liposomal-ICG resulted in significantly (p < 0.05) higher contrast-to-noise ratio compared to free ICG for extended periods of time, allowing visualization of hind limb and intracranial vasculature for up to 4 hours post-injection. *In vivo* comparisons demonstrated higher vessel conspicuity with liposomal-ICG-enhanced NIR-II imaging compared to NIR-I imaging.

## Introduction

Optical imaging in the near-infrared second window (NIR-II, 1000–1800 nm) holds considerable promise for *in vivo* imaging due to reduced light scattering by tissue, and increased penetration depth when compared to NIR imaging in the first window (NIR-I, 700–900 nm)^1–5^. As a result, there is growing interest in the development of fluorescent probes for NIR-II imaging^6^. Novel NIR-II imaging agents based on both macromolecular^7–9^ and nanoparticle carriers^1,10–13^ have been synthesized and evaluated in the pre-clinical setting. Nanoparticle platforms are particularly attractive for this purpose, enabling surface ligand attachment to generate targeted agents for molecular imaging applications, and carrying a large number of fluorescent molecules, enabling signal amplification at target sites. However, novel compounds constitute “new chemical entities” and face regulatory obstacles in clinical translation.

It was recently demonstrated that the FDA-approved agent, indocyanine green (ICG), fluoresces in the NIR-II window^14,15^. ICG in plasma or polar medium, when excited with a ~780 nm laser source, emits NIR-II fluorescence in the 1000–1400 nm window that can be detected using an InGaAs camera. *In vivo* NIR-II imaging studies using ICG enabled the visualization of deep structures with higher contrast-to-noise ratio (CNR) than in the NIR-I window. The availability of a high sensitivity NIR-II fluorophore opens the door for development of nanoparticle agents that can be easily tailored for molecular imaging applications.

While liposomal formulation of ICG (liposomal-ICG) has been investigated as a NIR-I agent^16–19^, its NIR-II fluorescence characteristics have not been reported to date. The association of ICG through hydrophobic interactions with lipids and polymers, and through electrostatic interactions with hydrophilic polyethylene glycol, potentially enhancing its stability and fluorescence, has been previously reported with liposomes and micelles^20,21^. Thus, encapsulating ICG in PEGylated liposomes could enhance its NIR-II fluorescence yield as well. In this work, we therefore investigated the NIR-II fluorescence properties of liposomal-ICG. We chose the liposome platform for its long clinical history and track-record of clinical translation. The NIR-II fluorescence properties of liposomal-ICG were determined in buffered saline and reconstituted bovine plasma using a NIR spectrophotometer. *In vitro* NIR-I and NIR-II imaging were performed in a tissue-mimicking Intralipid^®^ phantom. Finally, *in vivo* studies were performed in nude mice to study the performance of liposomal-ICG as a NIR-II imaging agent for visualization of deep structures. Imaging was performed in the hind limb, and in the brain within the intact skull. Comparisons were also made to cross-sectional imaging techniques (CT in the hind-limb and MRI in the brain).

## Results

### Physico-chemical characterization of liposomal-ICG

The mean particle size of liposomal-ICG was 89 ± 3 nm. The polydispersity index (PDI) was 0.15. The cumulative particle size distribution was 100% below 150 nm, 2% below 75 nm and 0% below 50 nm. The ICG content in the final formulation was 73 ± 4 µM and ICG: phospholipid molar ratio was 0.94. The NIR-I fluorescence intensity of liposomal-ICG measured over 80 days showed less than 10% drop in signal intensity demonstrating long-term stability of the formulation. In comparison, aqueous solutions of free ICG prepared in deionized water and buffered saline degraded relatively quickly with loss of 85% and 40% in water and buffered saline, respectively, by day 20. Neither preparation showed significant ICG loss in the first 24 hours.

### *In vitro* NIR fluorescence spectrum analysis

**Figure 1:**
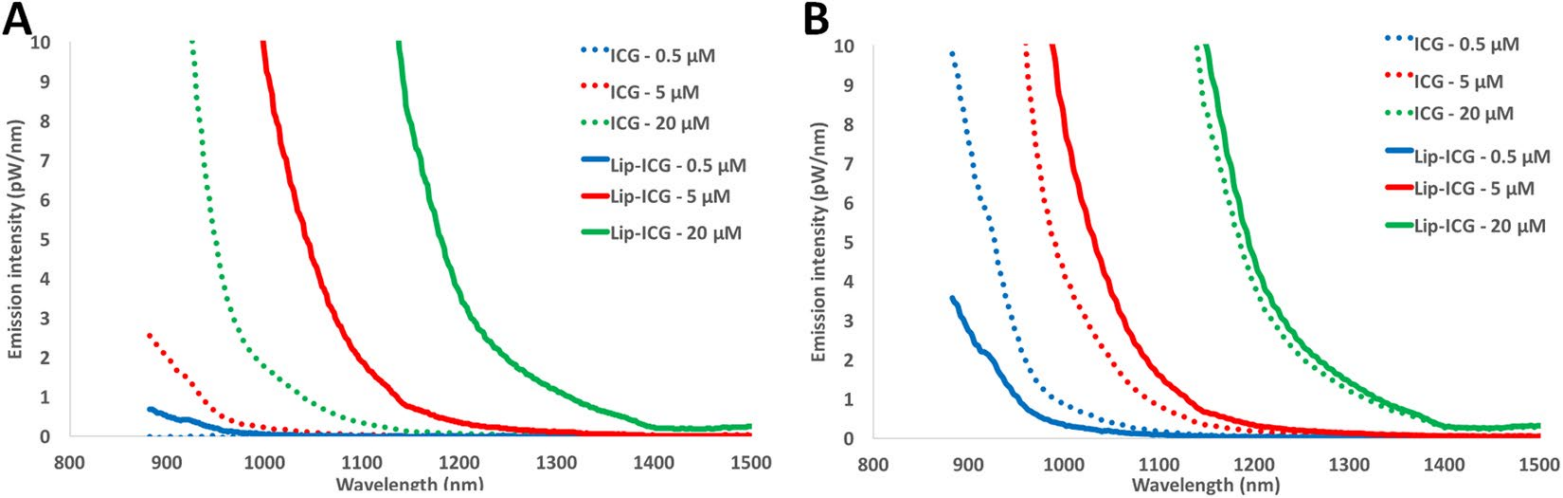
NIR-II emission spectra of liposomal-ICG (Lip-ICG) and free ICG. Lip-ICG and free ICG were diluted in either (**A**) phosphate buffered saline or (**B**) bovine plasma. Emission spectra were acquired for three ICG concentrations using an excitation wavelength of 782 nm.

In phosphate buffered saline (PBS), liposomal-ICG exhibited a NIR-II fluorescence emission spectrum similar to free ICG (Fig. 1A), but with far higher emission intensity. This enhancement was observed at all concentrations, and all wavelengths in the observed window. The enhanced NIR-II fluorescence of liposomal-ICG in PBS is similar to that in NIR-I and is attributed to interaction of ICG with components of liposome bilayer as reported previously^20^.

In plasma, free ICG exhibited a significantly higher fluorescence than in PBS, consistent with previous findings^14^. The higher fluorescence in plasma is attributed to interaction of ICG with plasma proteins. No such solvent-related differences in fluorescence emission were seen for liposomal-ICG. Liposomal-ICG did not exhibit such drastic changes in fluorescence intensity in plasma, and at all but the lowest concentration tested (0.5 µM), exhibited higher fluorescence intensity than free ICG in plasma (Fig. 1B).

### NIR-II imaging in Intralipid^®^ phantom

**Figure 2:**
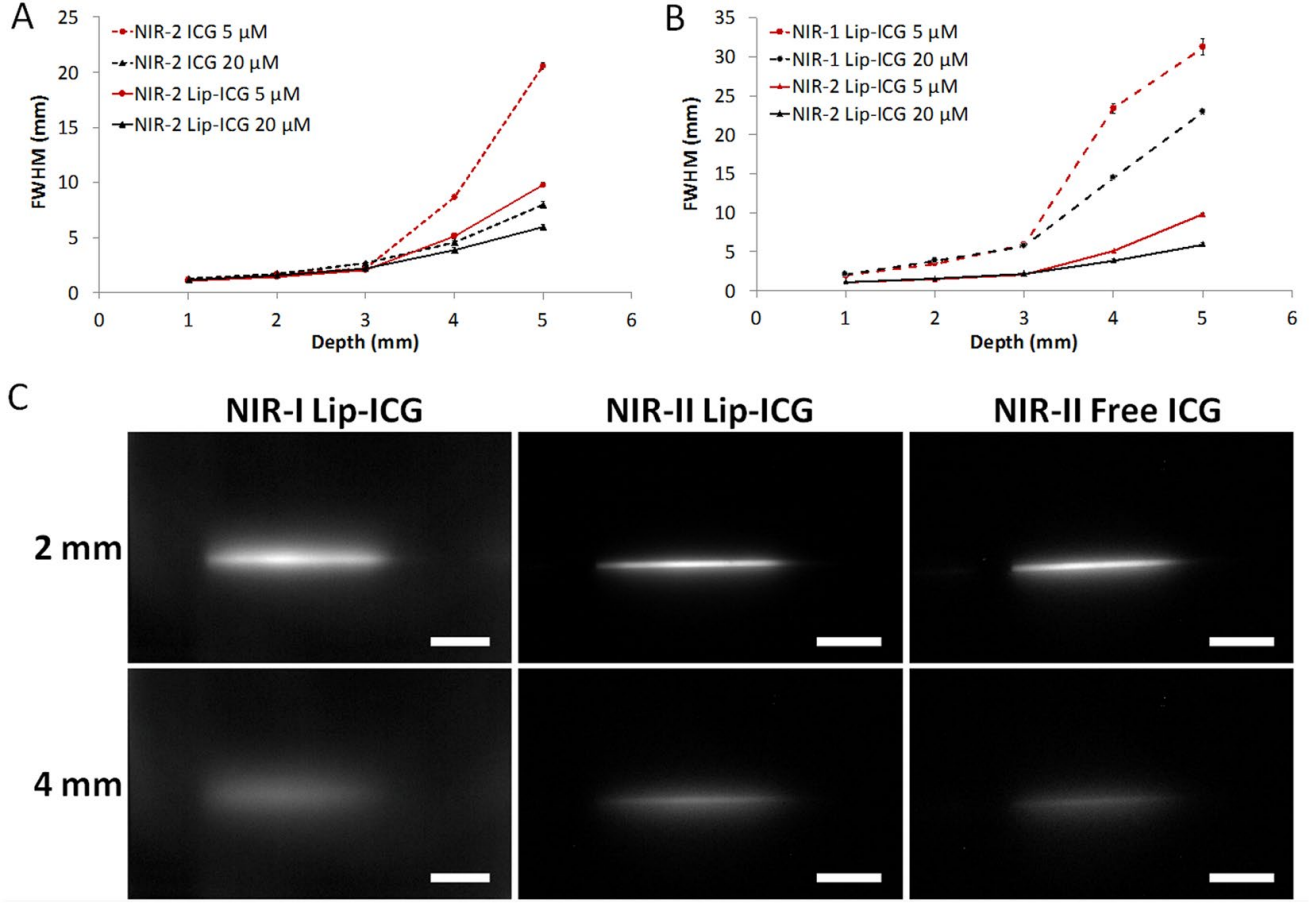
Determination of penetration depth for NIR-II imaging of liposomal-ICG (Lip-ICG) in an Intralipid^®^ phantom. (**A**) Comparison of Lip-ICG and free ICG in NIR-II window. Full-width-half-maximum (FWHM) of capillary glass tube filled with Lip-ICG or free ICG as a function of depth in a 1% Intralipid® phantom. FWHM for Lip-ICG were significantly different (p < 0.05) from free ICG at corresponding concentrations, at 4 and 5 mm depths. (**B**) Comparison of Lip-ICG in NIR-I and NIR-II window. FWHM of capillary glass tube filled with liposomal-ICG as a function of depth in Intralipid®. FWHM NIR-II window were significantly different (p < 0.05) from NIR-I window at their corresponding concentrations, at 4 and 5 mm depths. (**C**) Representative fluorescence images of glass capillary filled with either Lip-ICG or free ICG at depths of 2 and 4 mm in 1% Intralipid®. Scale bars represent 10 mm. Samples were prepared by diluting Lip-ICG or free ICG in plasma.

NIR-II imaging of tubes filled with either liposomal-ICG or free ICG in an Intralipid^®^ phantom indicated similar full width half maximum (FWHM) values for visualization of vascular structures at low penetration depths (Fig. 2A). The FWHM values are comparable for liposomal-ICG and free ICG at 5 and 20 µM concentrations up to 3 mm depth. Further, at 4 and 5 mm depths, NIR-II fluorescence images acquired with liposomal-ICG demonstrated significantly (p < 0.05) lower FWHM values compared to NIR-II images acquired with free ICG. NIR-II imaging of tubes filled with liposomal-ICG exhibited significantly (p < 0.05) lower values of FWHM when compared to NIR-I imaging (Fig. 2B), suggesting improved depth of penetration for visualization of deep vascular mimicking structures. Representative images of tubes filled with liposomal-ICG (NIR-I and NIR-II) and free ICG (NIR-II) at 2 and 4 mm depths (Fig. 2C) demonstrate improved tube conspicuity achieved in the NIR-II with liposomal-ICG (20 µM ICG concentration).

### *In vivo* NIR-II imaging

**Figure 3:**
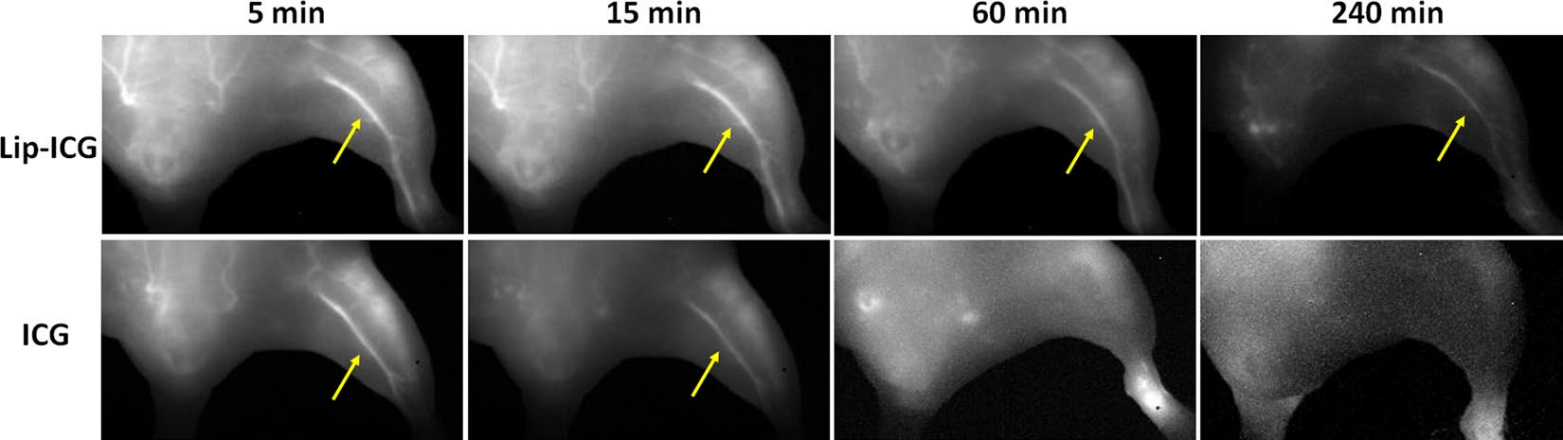
*In vivo* NIR-II imaging of hind limb vasculature. Representative coronal images of hind limb region demonstrating NIR-II imaging of vasculature at various time points after intravenous administration of either liposomal-ICG (top row) or free ICG (bottom row). At 60 min and 240 min, the femoral vessel (yellow arrow) is only visible in liposomal-ICG images.

**Figure 4:**
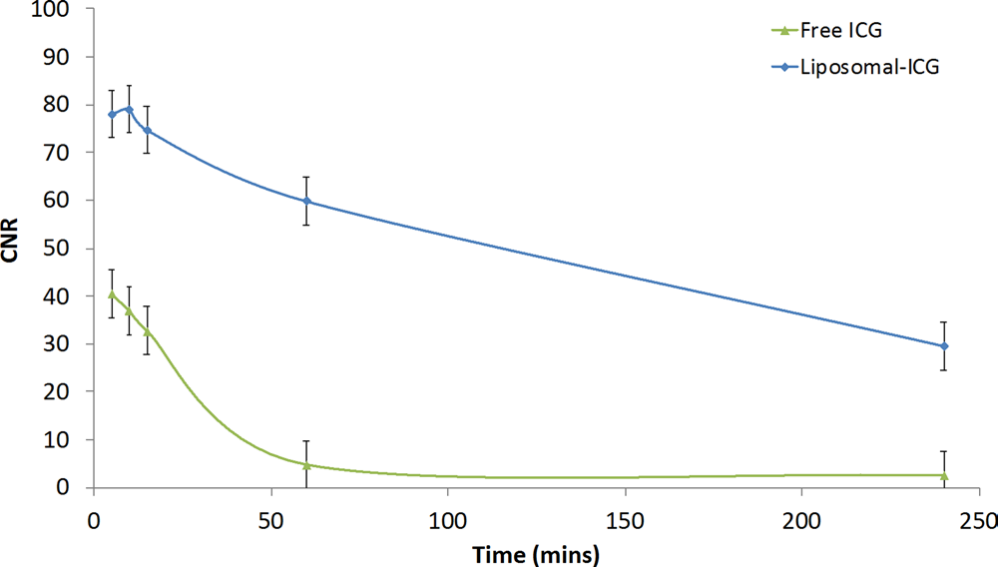
Contrast-to-noise ratio (CNR) for NIR-II imaging of hind limb vasculature. Normalized CNR values of femoral vessel in the hind limb region in NIR-II images acquired with either liposomal-ICG or free ICG. CNR values were determined for NIR-II images acquired at various time points after administration of contrast agent. CNR values were normalized to ICG dose (mg) per unit body weight (kg). CNR values for liposomal-ICG were significantly different (p < 0.05) from free ICG at all time points.

Nude mice were intravenously administered either liposomal-ICG or free ICG and imaged simultaneously using NIR-I and NIR-II cameras for up to 4 hours post-contrast injection. *In vivo* NIR imaging was performed to visualize blood vessels in the hind limb region and within the intact brain. In the hind limb, NIR-II imaging with liposomal-ICG enabled clear visualization of blood vessels that were also demonstrated by free ICG (Fig. 3). However, liposomal-ICG resulted in greater vessel conspicuity, and enabled prolonged visualization of vasculature for up to 4 hours, while with free ICG, blood vessels were not visualized beyond 60 mins. The contrast to noise ratio (CNR) was significantly higher (p < 0.05) for liposomal-ICG compared to free ICG at all time points (Fig. 4). At 15 minutes post-injection, the CNR values were 75 ± 5 with liposomal-ICG compared to 35 ± 5 with free ICG. At 4 hours post injection, free-ICG CNR drops to <5 while with liposomal-ICG the CNR remains significantly higher at 29 ± 5. The CNR for NIR-II imaging of the hind limb using liposomal-ICG was 2X higher than that for NIR-I imaging (Supplementary Information Figs S1 and S2).

**Figure 5:**
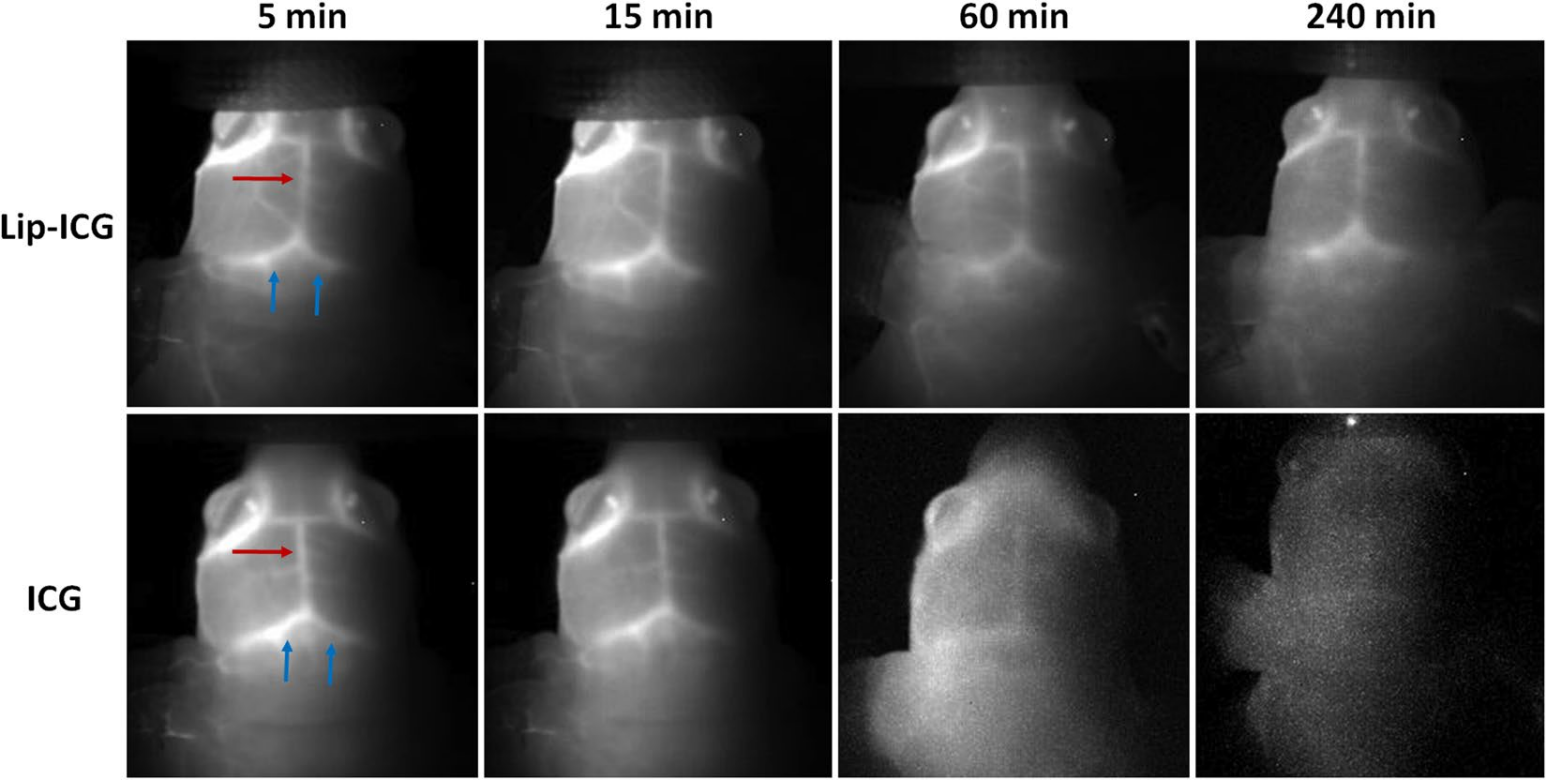
*In vivo* NIR-II imaging of brain vasculature through an intact skull. Representative coronal images of mouse brain demonstrating NIR-II imaging of vasculature at various time points after intravenous administration of either liposomal-ICG (top row) or free ICG (bottom row). At 60 min and 240 min, the transverse sinuses (blue arrows) and the sagittal sinus (red arrow) are only visible clearly in NIR-II images acquired with liposomal-ICG.

**Figure 6:**
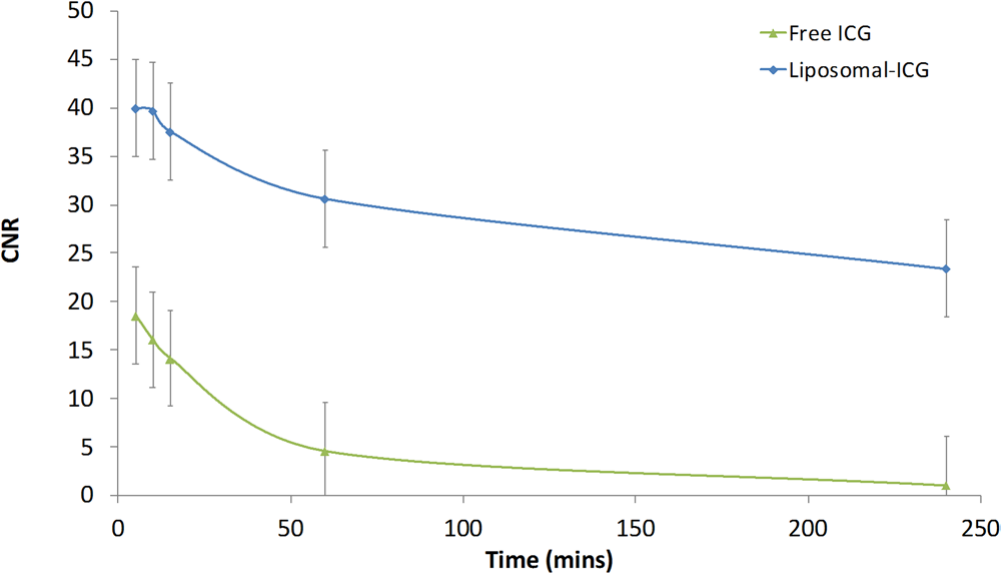
Contrast-to-noise ratio (CNR) for NIR-II imaging of blood vessels within the brain. Normalized CNR values of transverse sinus in NIR-II images acquired with either liposomal-ICG or free ICG. CNR values were determined for NIR-II images acquired at various time points after administration of contrast agent. CNR values were normalized to ICG dose (mg) per unit body weight (kg). CNR values for liposomal-ICG were significantly different (p < 0.05) from free ICG at all time points.

Similarly, in the brain, NIR-II imaging with liposomal-ICG enabled prolonged visualization of intracranial blood vessels, for up to 4 hours in comparison to free ICG (Fig. 5). Liposomal-ICG exhibited higher NIR-II signal and CNR compared to free ICG (Fig. 6). CNR values at 15 minutes post-injection were 37 ± 7 for liposomal-ICG and 15 ± 7 for free ICG. At 4 hours post-injection, CNR for free ICG drops to <1 (i.e. background noise levels) while with liposomal-ICG, it stays high at 24 ± 7. The CNR for NIR-II imaging in the brain was 2.5X the CNR for NIR-I imaging using liposomal-ICG (Supplementary Information Figs S3 and S4).

### Comparison with MRA and CTA

**Figure 7:**
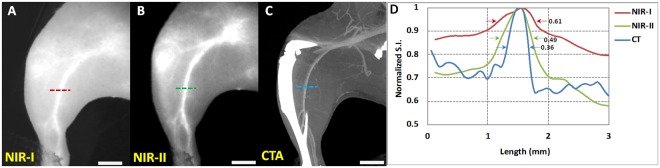
Comparison of NIR imaging and CT angiography (CTA) for visualization of hind limb vasculature. Representative coronal images of mouse hind limb region demonstrating the visualization of femoral vessel in (**A**) NIR-I, (**B**) NIR-II, and (**C**) CTA. (**D**) Normalized signal intensity profile across the cross-section of a femoral vessel in CTA, NIR-I and NIR-II window. Full width half maximum (FWHM) values are reported adjacent to each of the profiles. Note that the vessel profile in CT matches closer to the profile in NIR-II than in NIR-I. Scale bars represent 5 mm.

**Figure 8:**
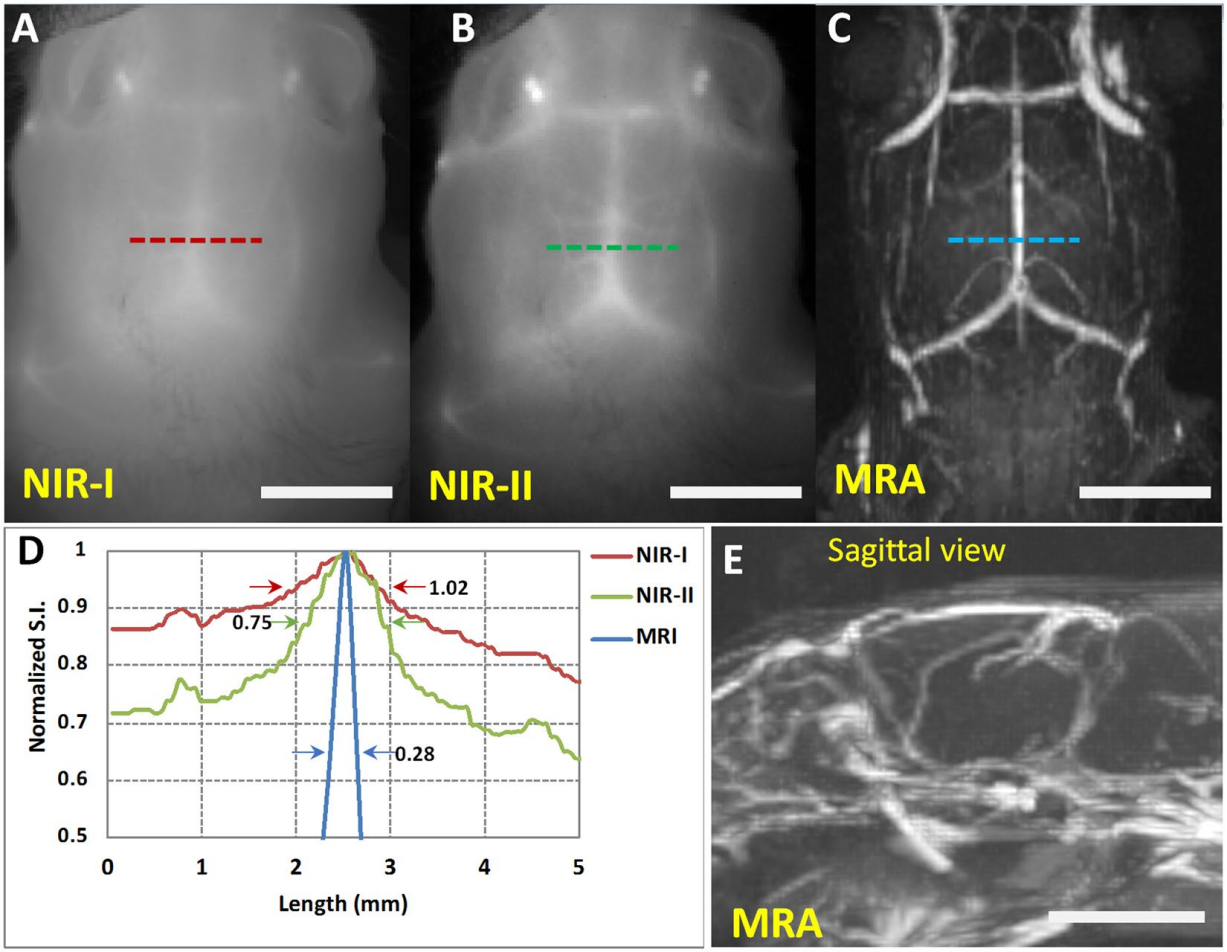
Comparison of NIR imaging and MR angiography (MRA) for visualization of intracranial vasculature. Representative coronal images of mouse brain demonstrating the visualization of intracranial vessels in (**A**) NIR-I, (**B**) NIR-II, and (**C**) MRA. (**D**) Normalized signal intensity profile across the cross-section of a sagittal sinus in NIR-I and NIR-II imaging window. Full width half maximum (FWHM) values are reported adjacent to each of the profiles. (**E**) Sagittal view of mouse brain demonstrating the depth of intracranial vessels from the top. All images were acquired in the same animal. Scale bars represent 5 mm.

NIR images were compared with standard cross-sectional images for the visualization of vascular structures. Contrast-enhanced CT imaging was used as the comparator for imaging hind limb vasculature. Contrast-enhanced MR imaging was used as the comparator for imaging intracranial vasculature. MRI was chosen for brain imaging because the presence of the skull causes streak artifacts on CT images, making it difficult to visualize vessels in the immediate vicinity of the skull. Contrast-enhanced NIR images were acquired using liposomal-ICG. MR angiography (MRA) images were acquired using a long circulating liposomal-Gd blood-pool contrast agent whereas CT angiography (CTA) images were acquired using a long circulating liposomal-iodine blood-pool contrast agent. NIR-II imaging using liposomal-ICG yielded vessel cross-sectional profiles that were closer to the cross-sectional comparator images than NIR-I imaging, in both hind limb and intracranial vasculature (Figs 7 and 8). In hind limb imaging, the full width half maximum (FWHM) values were 0.6 mm for NIR-I imaging, 0.46 mm for NIR-II imaging and 0.36 for CTA. In the brain, the FWHM values were 1.02 mm for NIR-I imaging, 0.75 mm for NIR-II imaging and 0.28 for MRA.

## Discussion

Real-time near infrared imaging has applications in lymphangiography, and intraoperative imaging including the detection of occult vascular structures, sentinel lymph nodes and tumor margin detection^22–29^. The second near-infrared window (NIR-II, 1000–1800 nm) has recently been recognized as a superior alternative to the traditionally used NIR-I (780–900 nm) window^2,4,7,14,16^. NIR-II cameras are based on compound semiconductor detector elements, usually InGaAs, because of their lower bandgap and higher sensitivity at the lower energy levels of the NIR-II window. This is advantageous because, in contrast to NIR-I imaging, which is the current clinical standard for fluorescence imaging, the longer wavelengths of the NIR-II window are less affected by absorption and scattering effects^30–32^, thus improving signal-to-noise ratio (SNR) and depth of tissue penetration^10,33^, potentially allowing for improved visualization of deep structures. Limitations to this approach however, include the lack of suitable fluorescent dyes that are approved for clinical use. Several novel molecules have been investigated for this purpose, but are still a long way from regulatory approval. However, we and others^14,15^ have recently shown that the clinically approved dye indocyanine green (ICG), exhibits significant emission in the NIR-II window, and could be adapted to NIR-II use. The availability of an FDA-approved agent combined with increasingly available InGaAs detector cameras is likely to accelerate the clinical translation of NIR-II fluorescence imaging for lymphangiography, and intra-operative procedures in both general and oncologic surgery.

However, rapid clearance from systemic circulation and the inability to target specific cells or receptors of interest limits the utility of free ICG. Nanoparticle-based formulations of ICG that exhibit NIR-II fluorescence similar to the free molecule could overcome these limitations and expand the utility of ICG-based NIR-II imaging into vascular and molecular imaging. In this work, we investigated the NIR-II imaging characteristics of a liposome-based ICG agent (liposomal-ICG). *In vitro* analysis of fluorescence spectra demonstrated that liposomal-ICG does indeed exhibit NIR-II fluorescence. While free ICG exhibited strong NIR-II fluorescence only in a plasma medium, liposomal-ICG demonstrated strong NIR-II fluorescence in both aqueous buffer and plasma medium, consistent with lipid interaction being the dominant mechanism for ICG fluorescence.

*In vitro* stability analysis demonstrated prolonged shelf-life stability of NIR fluorescence for liposomal-ICG compared to free ICG solution. While free ICG solutions degraded by up to 85% over 20 days, liposomal-ICG solutions only degraded marginally over the same period, and by no more than 10% over 80 days.

*In vivo* NIR-II imaging using liposomal-ICG demonstrated improved visualization of deep features compared to ICG, consistent with the vascular retention of liposomes. The long circulating property of liposomal nanoparticles enabled prolonged *in vivo* visualization of vasculature compared to free ICG. The agent enabled visualization of vasculature in both the hind limb region and in the brain, through the intact skull. The visualization of deep vascular structures in NIR-II window may be improved by employing alternate hardware. For instance, the use of a higher quantum efficiency NIR-II camera^7,15^ and higher power laser source^2^ may improve conspicuity of deep vascular structures. The kinetics of liposomal-ICG in these mouse studies suggest a circulation half-life on the order of a few hours, significantly faster compared to PEGylated liposomes (18–24 hours)^34–36^. This accelerated clearance is consistent with *in vivo* leakage of the ICG from the liposomes. Such leakage was not observed *in vitro* as demonstrated by long term stability of liposomal-ICG. However, clearance of both free ICG and PEGylated liposomes occurs predominantly via the liver^25,34,37,38^, suggesting that even if such leakage were occurring, the toxicity profile of ICG is not likely to be substantially different from that of liposomal-ICG. No acute toxicity was noted in the current studies, however, the complete toxicity profile of liposomal-ICG will need to be assessed in future work.

There are currently no FDA-approved agents for NIR-II; however, studies have clearly demonstrated that the second NIR window (NIR-II) exhibits better tissue transparency characteristics compared to NIR-I window. Novel molecules for NIR-II imaging are in development, but are likely years from clinical approval. The recent discovery that clinically approved ICG is a satisfactory NIR-II dye, with performance comparable to, or better than the novel molecules, constitutes a significant step towards the adoption of NIR-II in the clinic. However, the relatively short circulation half-life of ICG, and the uncertain fluorescent intensity due to variable binding to plasma proteins limits longitudinal imaging, and quantification. The development of liposomal-ICG addresses both these concerns, stabilizing the fluorescent yield by direct interaction of ICG with the lipid bilayer, and enabling long circulation. Finally, liposomal encapsulation of ICG opens the door for development of targeted imaging agents, suitable for deep tissue and intra-operative molecular imaging^39,40^.

## Materials and Methods

### ICG solutions

ICG solutions were prepared by dissolving ICG in either deionized (DI) water, phosphate buffered saline (PBS), histidine buffered saline (HBS) or citrated bovine plasma. Solutions were used for *in vitro* and *in vivo* studies within 3 hours of preparation.

### Fabrication of liposomal-ICG

1,2-Dipalmitoyl-sn-Glycero-3-Phosphatidylcholine (DPPC) and Cholesterol (Chol) were purchased from Lipoid GmbH (Ludwigshafen, Germany). N-(carbonyl-methoxy polyethylene glycol 2000)-1,2-Distearoyl-sn-Glycero-3 phosphoethanolamine (mPEG2000-DSPE) was purchased from Genzyme Pharmaceuticals (Cambridge, MA, USA). Whatman Nuclepore polycarbonate track-etch membranes of 100 nm and 400 nm pore sizes were purchased from Fisher Scientific (Waltham, MA, USA). Indocyanine green (ICG) was purchased from Sigma-Aldrich (St. Louis, MO, USA).

Liposomal-ICG was prepared using passive encapsulation^41–43^. Briefly, lipids (DPPC, Chol, mPEG2000-DSPE) were dissolved at 87:10:3 molar ratio in ethanol at 60–65 °C. ICG dissolved in ethanol (10 mg/mL) was added to lipids to achieve a 100 µM final ICG concentration in liposomal solution. The dissolved lipids were hydrated with histidine buffered saline (HBS) (10 mM Histidine, 140 mM NaCl, ~pH 7.6) at 60–65 °C to achieve a total lipid concentration of 100 mM. The hydrated lipid solution was extruded sequentially through 400 nm (4 passes) followed by 100 nm (5 passes) Nuclepore membranes at 60–65 °C using a high-pressure extruder (Northern Lipids, Vancouver, BC, Canada) to form liposomes of desired size.

The liposomal suspension was dialyzed against Histidine-buffered saline (HBS) using 300 kDa molecular weight cutoff membranes (Spectrum Laboratories Inc., CA, USA,) to remove un-encapsulated ICG and ethanol.

### Physicochemical characterization

The mean particle size of liposomes was determined using a Dynamic Light Scattering (DLS) instrument (Brookhaven Instruments Corp., Holtsville, NY, USA). Total phospholipid content of liposomal formulations was determined by measuring total phosphorus using inductively-coupled plasma atomic emission spectroscopy (ICP-AES)^44^. ICG concentration was determined by dissolving liposomal-ICG in dimethyl sulfoxide (DMSO) (1000x dilution) and measuring fluorescence (ex: 785 nm, em: 810–830 nm) using a Pioneer camera (Basler, Ahrensburg, Germany), with a Sony ICX625 CCD sensor 2456 × 2058 pixels (5 Mpixels), fitted with 810–830 nm bandpass filter. The fluorescence intensity was converted into ICG concentration using a calibration curve that was generated with free ICG dissolved in DMSO (concentration range: 2–200 nM).

The *in vitro* stability of liposomal-ICG was studied by monitoring ICG fluorescence. The formulations were monitored for over 80 days. Free ICG solutions prepared in deionized (DI) water, phosphate buffered saline (PBS), and histidine buffered saline (HBS) were used as controls. Liposomal-ICG and free ICG solutions were stored at 4–8 °C in sealed containers, wrapped in aluminum foil to eliminate light exposure. Liposomal-ICG and free ICG solutions were diluted in HBS (100x dilution) for measurement of fluorescence intensity.

### *In vitro* NIR fluorescence spectrum analysis

Fresh dilutions of liposomal-ICG and free ICG were prepared at ICG concentrations of 0.5, 5 and 20 µM in PBS and bovine plasma (Sigma-Aldrich, St. Louis, MO, USA). The NIR-II fluorescence emission spectra of the solutions were acquired using a NS3 NanoSpectralyzer (Applied NanoFluorescence LLC., Houston, TX, USA). The emission spectra (900–1500 nm) were collected for 500 cycles of 10 ms each using an excitation wavelength of 782 nm.

### NIR-II imaging in Intralipid^®^ phantom

The NIR-II characteristics of liposomal-ICG were evaluated *in vitro* in a tissue mimicking phantom. All experiments were conducted in triplicates. A 1% Intralipid® solution was prepared by diluting 20% Intralipid® (Baxter Healthcare Corp., Deerfield, IL, USA) in deionized water. A cylindrical reservoir filled with 1% Intralipid® solution was positioned on a stage. The height of the stage was adjusted with a micrometer driver (Mitutoyo 151-411ST). A capillary glass tube (OD = 1.55 mm/ID = 1.0 mm) was immersed in the Intralipid® solution. The capillary tube filled with either free ICG or liposomal-ICG was imaged at depths from 1 to 5 mm from the top surface.

An in-house assembled system that enabled simultaneous imaging in NIR-I and NIR-II window was used for image acquisition^14^. The imaging hardware consisted of the following components (all acquired from ThorLabs, Newton, NJ, USA): 785 nm laser diode, LD785-SE400 with a TLCDM9 mount, a TEC controller TED200C, laser driver LDC205C, and a beam expander BE05M-B. The laser diode was driven per the manufacturer’s recommended settings of 475 mA and 2V, 400 mW optical power. In order to acquire NIR-I images, a gold-plated mirror (Mirror FS 1/10 wave gold 100 SQ, Edmund Optics, NJ, USA) was utilized to reflect the image from the phantom to the camera lens. Angular difference between collected images, ϕ, was 12 ± 0.2°. A Raptor-Ninox 640 SWIR camera (Phoenix Engineering Inc., Berkeley Lake, GA, USA) with a 1100 nm long pass filter (ThorLabs Inc., Newton, NJ, USA) was used to acquire images (50 frames at 40 ms each) in the NIR-II window. Images in the NIR-I window were captured (50 frames at 40 ms each) using a Pioneer camera (Basler, Ahrensburg, Germany), with a Sony ICX625 CCD sensor 2456 × 2058 pixels (5 Mpixels), fitted with 810–830 nm bandpass filter. Fluorescence images of the glass capillary tube filled with liposomal-ICG or free ICG solutions (5 and 20 µM concentration) prepared in bovine plasma were acquired and analyzed. Due to scattering of the emitted fluorescence, acquired images of the capillary tubes were broadened. Full width half maximum(FWHM) measurements of the broadening were used to characterize the visualization as a function of depth.

### *In vivo* NIR-II imaging

All *in vivo* procedures were performed under a protocol approved by the Baylor College of Medicine Institutional Animal Care and Use Committee. Studies reported in this paper are in accordance with the NC3RS ARRIVE guidelines. Studies were performed in female NCr nude mice (22 ± 2 g, 8 ± 2 weeks old) from Taconic Biosciences. Imaging was performed in the hind-limb area and the brain to study the visualization of vascular structures with and without bone interference. Simultaneous imaging in NIR-I and NIR-II window was performed using the prototype described above.

Mice were anesthetized using 2% isoflurane in an induction chamber and then maintained on 1–1.5% isoflurane delivered by nose cone. The tail vein was catheterized for injection of the contrast agent. For hind limb imaging, the animals were secured to a platform in the supine position with the ventral side facing the camera and laser source. The hind limb region of the animal was exposed to laser source (785 nm) and baseline pre-contrast NIR-I and NIR-II images were acquired using the respective cameras. The laser source power density at the imaging stage was 55 mW/cm^2^, well below the limit of 329 mW/cm^2^ established^45^ for safe exposure. ICG injected animals were imaged simultaneously using NIR-I and NIR-II cameras. Mice (n = 3 per group) were injected with a bolus of either ICG solution in PBS (0.72 ± 0.04 mg/kg) or liposomal-ICG (0.42 ± 0.03 mg/kg of ICG) via the tail vein catheter. The bolus injection was followed by a 100 µl saline flush, the entire injection procedure taking <5 seconds. Images were captured during injection and then at 5, 10, 15, 60, and 240 minutes post-injection.

For brain vascular imaging, animals were positioned in the prone position, the head was exposed to the laser source to acquire the baseline pre-contrast NIR images, and procedures identical to those described for hind limb imaging were performed for dynamic imaging of the head region.

### MRI and CT angiography

The visualization of vascular structures in liposomal-ICG enhanced NIR imaging was compared to conventional, high-resolution cross-sectional imaging. In the hind limb, NIR images were compared to contrast-enhanced CT images; while in the brain, NIR images were compared to contrast-enhanced MRI. Hind limb and brain vascular imaging were performed in separate groups of animals (n = 3 in each group). First, the animals were administered liposomal-ICG and simultaneously imaged by NIR-I and NIR-II systems as described above. The brain vascular imaging group was then imaged by MRI, and the hind limb vasculature group by CT as described below.

For brain imaging studies, animals were administered a long circulating blood pool liposomal-Gadolinium (liposomal-Gd, 0.1 mmol Gd/kg) contrast agent for *in vivo* magnetic resonance angiography (MRA) prepared using methods described previously^44,46^. Imaging was performed on a 1 Tesla permanent MRI scanner (M2 system, Aspect Imaging, Shoham, Israel). This contrast agent is optimized for maximum contrast-to-noise ratio at this field strength, and enables facile high resolution imaging^44^. A 25 mm volume coil was used for brain imaging. Animals were anesthetized using 3–4% isoflurane, placed on the MRI animal bed and then maintained at 1–1.5% isoflurane delivered via nose cone. Respiratory rate was monitored using a pneumatic pressure pad placed underneath the animal. Imaging was initiated within 15 minutes after injection of contrast agent. Images were acquired using a T1-weighted (T1w) gradient echo (GRE) sequence with the following scan parameters: echo time (TE) = 3.5 ms, repetition time (TR) = 20 ms, slice thickness = 0.3 mm, field of view = 54 mm, number of slices = 80, matrix = 180 × 180, acquisition plane = coronal, number of excitations = 1, scan time ∼6 min. These parameters resulted in an isotropic voxel size of 0.3 mm. Four scans were acquired and externally averaged.

For hind limb imaging studies, animals were administered a liposomal-iodine (liposomal-I, 1.1 g I/kg) contrast agent for computed tomography angiography (CTA) prepared using previously published methods^41,47^. CT imaging was performed on a small animal micro-CT scanner (Inveon, Siemens Inc., Knoxville, TN, USA). Animals were anesthetized using 3–4% isoflurane, positioned on the CT cradle and then maintained at 1–1.5% isoflurane delivered via nose cone. Respiratory rate was monitored by a pneumatic pressure pad placed underneath the animal. Imaging was initiated within 15 minutes after injection of contrast agent. Images were acquired using the following scan parameters: 70 kVp, 0.5 mA, 850 ms X-ray exposure, 540 projections, 35 µm reconstructed voxel size, ~20 min scan time.

### Data and statistical analysis

Analysis of *in vitro* and *in vivo* images was performed using FIJI software (v 2.0.0-rc-49/1.51 f, https://imagej.net/Fiji/Downloads). Image resolution was 2456 × 2058 pixels for NIR-I camera and 640 × 512 pixels for NIR-II camera. For *in vivo* data analysis, the NIR-I images were 4X-binned to enable comparison of NIR-I and NIR-II images at similar spatial resolution. Analysis was performed on an averaged intensity image generated from a stack of 500 images. Quantitative analysis of signal enhancement was performed based on measurements of signal intensity (*SI*) in selected regions of interest (ROIs) similar to methods described previously^14^. Signal-to-noise ratios (*SNR*) were calculated as mean signal intensity in ROIs divided by noise, where noise was represented as the standard deviation (*SD*) in an ROI drawn in tissue that was not illuminated by the excitation beam. Contrast to noise ratios (*CNR*) were calculated as the difference between SNR in the vessel and SNR in the region proximal to the vessel and normalized to ICG dose (mg ICG/kg body weight) facilitating comparison of image quality for mice injected with either liposomal-ICG or free ICG. Statistical analysis was performed using a Kruskal-Wallis test. A p-value less than 0.05 was considered statistically significant.

## Electronic supplementary material

Supplementary Information

## Data Availability

The datasets generated and/or analyzed during the current study are available from the corresponding author on reasonable request.
