# Supplementary material for: NIR-II fluorescence imaging using indocyanine green nanoparticles
Source: Sci Rep. 2018 Sep 27;8:14455. doi: 10.1038/s41598-018-32754-y (PMC6160486; doi:10.1038/s41598-018-32754-y)
Supplement: Supplementary file 1 — Supplementary Information [file 41598_2018_32754_MOESM1_ESM.pdf]

NIR-II fluorescence imaging using indocyanine green nanoparticles

Rohan Bhavane, Zbigniew Starosolski, Igor Stupin, Ketan B. Ghaghada, Ananth Annapragada

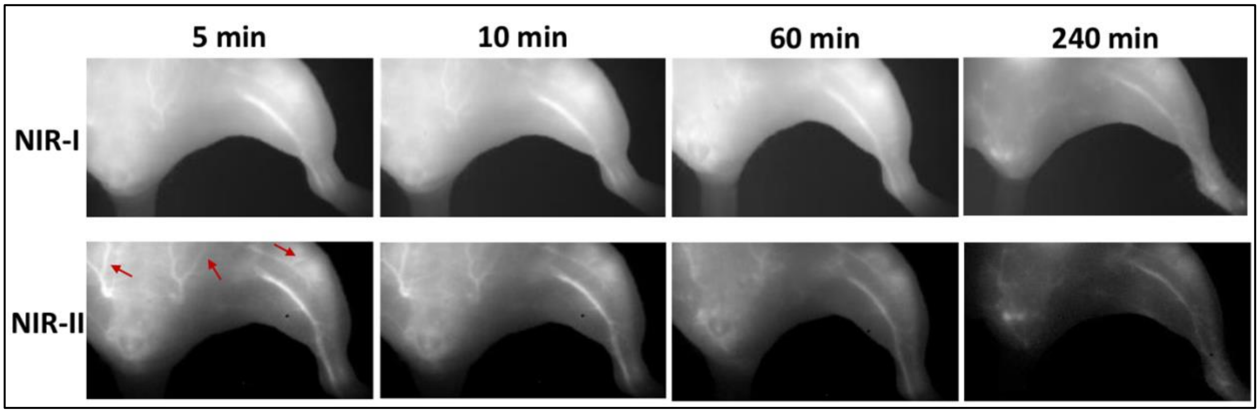

Figure S1. Comparison of NIR-I and NIR-II images of hind limb vasculature obtained using liposomal-ICG. Arrows point to vascular features clearly/only visible in NIR-II images.

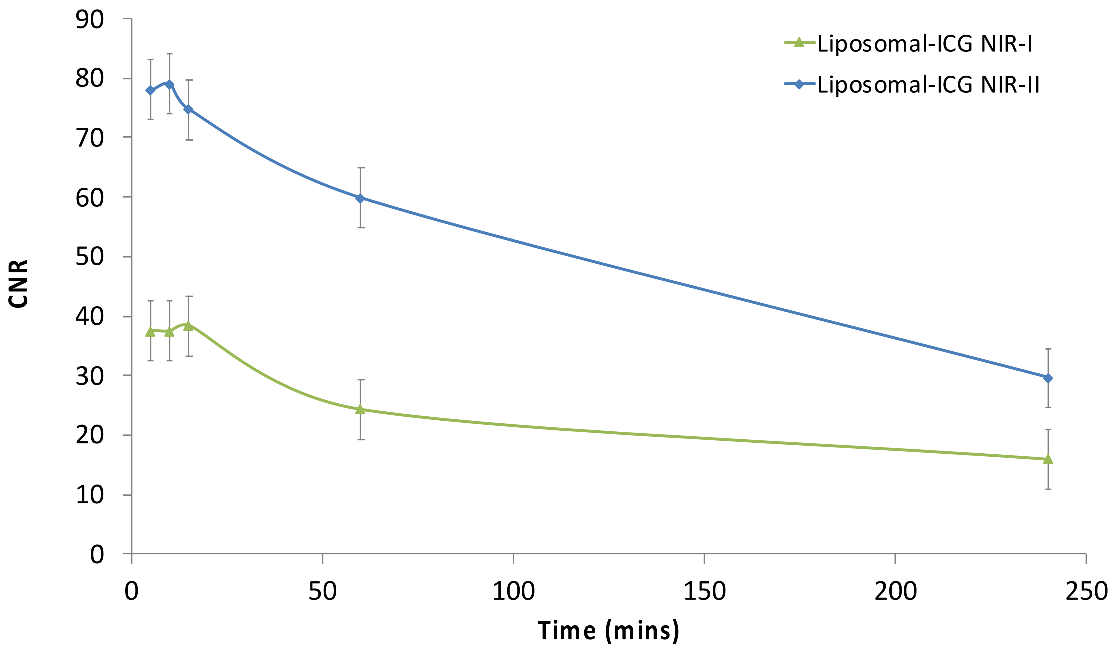

Figure S2. Contrast-to-noise ratio (CNR) for femoral vessel in the hind limb region in NIR-I and NIR-II images acquired with liposomal-ICG. CNR values were normalized to ICG dose (mg) per unit body weight (kg). CNR values for liposomal-ICG were significantly different ($p < 0.05$) from free ICG at all time points.

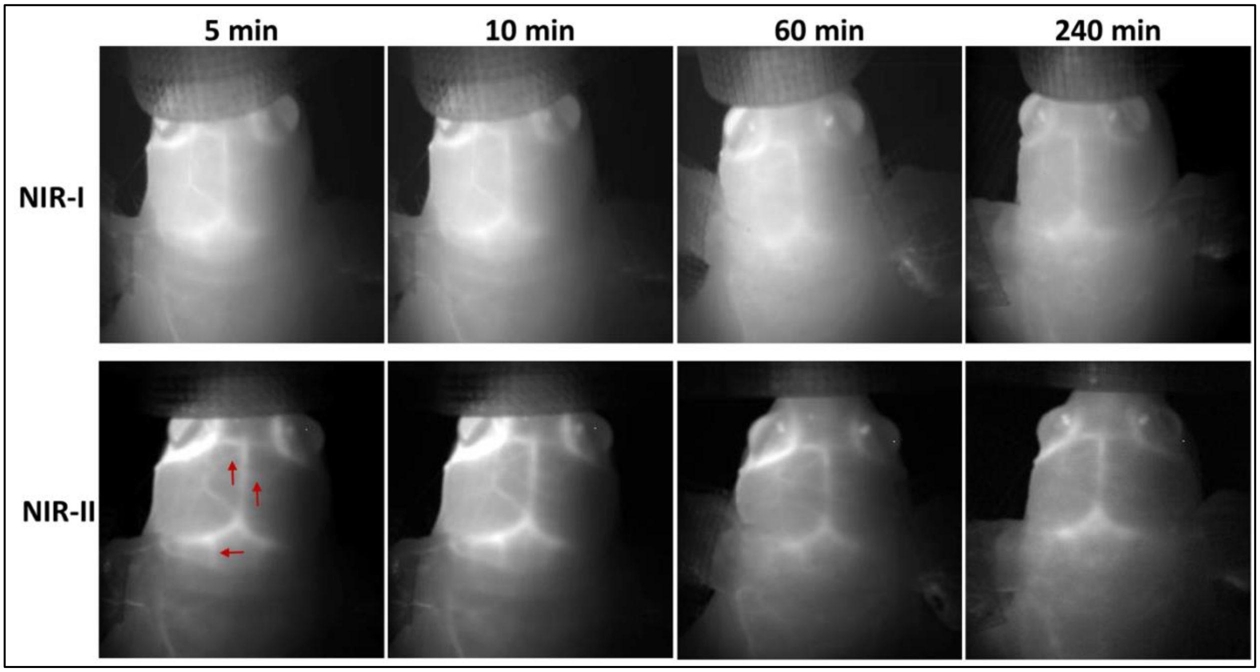

Figure S3. Comparison of NIR-I and NIR-II images of brain vasculature acquired using liposomal-ICG. Arrows point to vascular features clearly/only visible in NIR-II images.

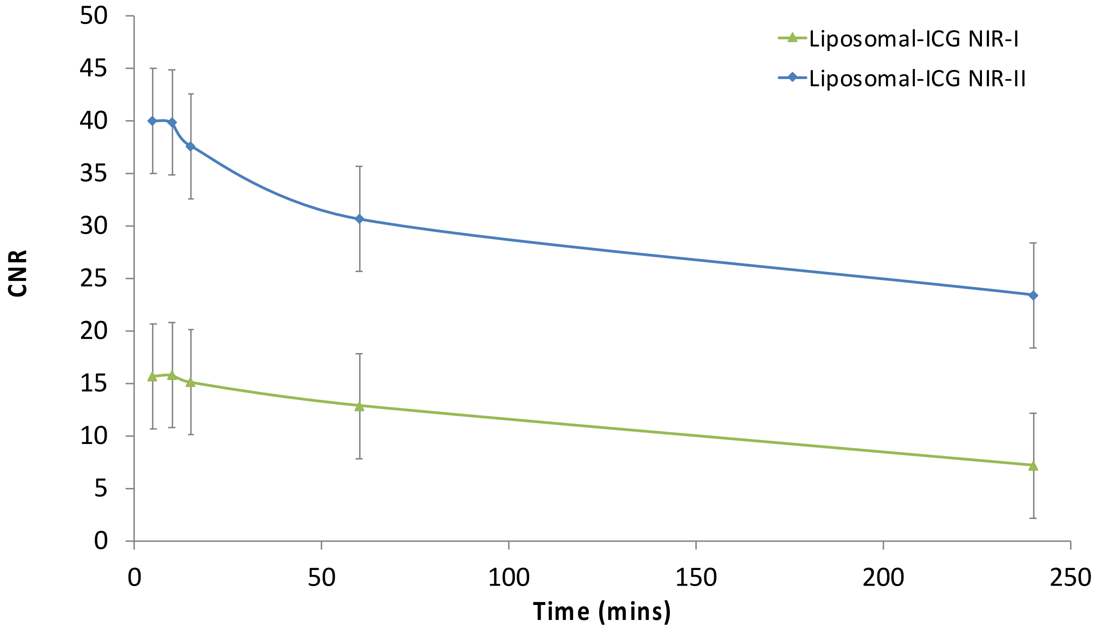

Figure S4. Contrast-to-noise ratio (CNR) for brain vessel (transverse sagittal sinus) in NIR-I and NIR-II images acquired with liposomal-ICG. CNR values were normalized to ICG dose (mg) per

unit body weight (kg). CNR values for liposomal-ICG were significantly different ($p < 0.05$) from free ICG at all time points.
